# Database of RNA binding protein expression and disease dynamics (READ DB)

**DOI:** 10.1093/database/bav072

**Published:** 2015-07-25

**Authors:** Seyedsasan Hashemikhabir, Yaseswini Neelamraju, Sarath Chandra Janga

**Affiliations:** ^1^Department of Biohealth Informatics, School of Informatics and Computing, Indiana University Purdue University, 719 Indiana Ave Ste 319, Walker Plaza Building, Indianapolis, IN, 46202, USA,; ^2^Center for Computational Biology and Bioinformatics, Indiana University School of Medicine, 5021 Health Information and Translational Sciences (HITS), 410 West 10th Street, Indianapolis, IN, 46202, USA, and; ^3^Department of Medical and Molecular Genetics, Indiana University School of Medicine, Medical Research and Library Building, 975 West Walnut Street, Indianapolis, IN, 46202, USA

## Abstract

RNA Binding Protein (RBP) Expression and Disease Dynamics database (READ DB) is a non-redundant, curated database of human RBPs. RBPs curated from different experimental studies are reported with their annotation, tissue-wide RNA and protein expression levels, evolutionary conservation, disease associations, protein–protein interactions, microRNA predictions, their known RNA recognition sequence motifs as well as predicted binding targets and associated functional themes, providing a one stop portal for understanding the expression, evolutionary trajectories and disease dynamics of RBPs in the context of post-transcriptional regulatory networks.

**Database URL:** READ DB is freely available on the web at http://darwin.soic.iupui.edu/ with all major browsers supported.

## Introduction

RNA Binding Proteins (RBPs) have a primary role in the post-transcriptional regulation of genes by adding an extra level of plasticity in controlling gene expression (1). They form dynamic Ribonucleoprotein (RNP) complexes and control various stages in the metabolism of RNA. The process of binding to RNA is mediated by RNA binding domains such as the RNA Recognition Motif (RRM), K homology domain (KH) domain etc. (2). Individual RBPs contain multiple domains that can independently bind to RNA. In recent years, several methods like SELEX, CLIP, PAR-CLIP, iCLIP, RNA compete have been developed to identify RNA bound proteome which lead to an addition of novel RBPs to those previously known (3–5). Here, we present READ DB which is a unified resource of ∼1350 RBPs in humans curated from recent experimental studies. We report multiple properties of RBPs including gene summary information from NCBI, tissue-wide transcript and protein expression levels from the Human Body Map (6) and Human Proteome (7) Projects respectively, evolutionary conservation, physical high-confidence protein interactions from BioGRID (8), disease associations from MalaCards (9), miRNA target predictions for RBPs, RNA recognition sequence motifs and their predicted binding targets as well as enriched processes and pathways in the human genome, in the form of an easy to navigate and accessible resource for researchers working on specific groups of RBPs.

## Materials and methods

### Data collection

**Figure 1.**
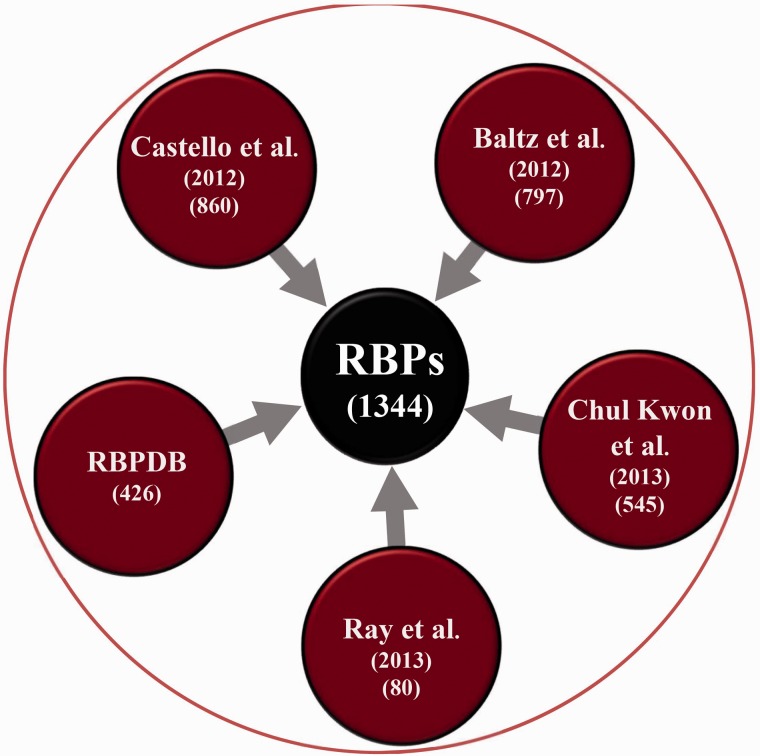
Schematic showing the number of RBPs collected from various experimental studies.

A non-redundant and curated list of 1344 RBPs in the human genome was constructed from various experimental sources (10) as described later (See Figure 1):

- mRNA interactome of HeLa cell (3),
- mRNA bound proteome identified from photoreactive nucleoside-enhanced UV crosslinking and oligo(dT) affinity purification approach (4),
- Human orthologs of proteins identified in mouse embryonic stem cells to be bound to RNA (5),
- RBPs screened in a RNA compete study (11),
- RBPs reported in RBPDB (12).

**Figure 2.**
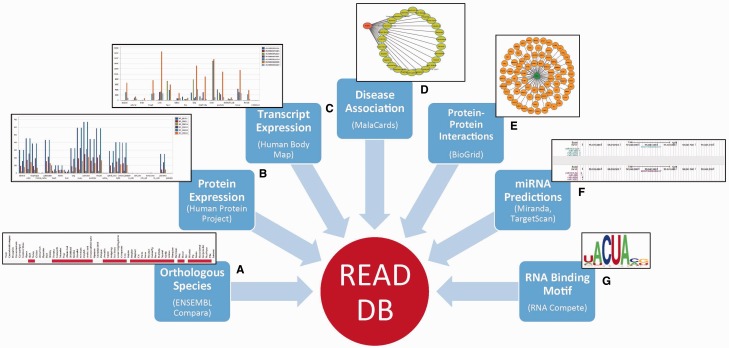
Integration of data from multiple sources in READ DB.

Each RBP in the database can be queried for or is associated with the following levels of information as summarized in Figure 2

#### (A) Summary

This section provides for each RBP, its synonyms from HGNC annotations, Ensembl accession ID and a description from the NCBI Refseq. We have also included subcellular localization from Uniprot database (13).

#### (B) Domain Information

We extracted domain information for every RBP from Pfam database (14) and present a visualization of its domain architecture using Prosite MyDomains (15).

#### (C) Evolutionary Conservation

The orthologs of human RBPs in 62 different species are extracted from Ensembl (v73) compara (16). These species come from different taxonomic groups namely—Fungi, Chordates, Reptiles, Aves, Amphibians, Mammals, Insects, Rodents and Primates. We represent the extent of conservation as a heatmap as well as a phylogenetic gene tree for each queried RBP. Phylogenetic trees are imported from Ensembl and users can also access the gene tree and alignment files for the specific RBP being queried.

#### (D) Protein Expression

The expression levels of all the protein isoforms corresponding to each RBP gene are extracted from the human proteome map for 24 different tissues (7).

#### (E) Transcript Expression

Transcripts annotated as ‘protein_coding’ in Ensembl are extracted and their expression levels across 16 different tissues are obtained from the Human Body Map (6).

#### (F) Disease Association

Diseases associated with each RBP along with their relevance scores are obtained from the Malacards database (9).

#### (G) Protein–Protein Interactions

Protein interactions for RBPs are retrieved from BioGRID database (8). We report the interacting partner of each RBP as well as their synonyms, evidence code for the reported protein interaction along with the supporting publications.

#### (H) MicroRNA Predictions

MicroRNAs (miRNAs) targeting the 3′UTR of each protein-coding transcript of a given RBP are predicted using Miranda (17) and TargetScan (18) algorithms. Miranda predicts microRNA targets based on a three phase method that incorporates sequence-matching to estimate the complementarity between a miRNA and a potential target gene to give a score (S). In contrast, TargetScan searches for the presence of conserved 8mer and 7mer sites that match the seed region of each miRNA. The prediction efficacy is calculated as context +score of the sites which is the sum of site-type contribution, 3′ pairing contribution, local AU contribution and position contribution. To include all potential factors that can facilitate a stronger bond between miRNA and its target RBP transcript, we included only those miRNAs that are predicted at a defined recommended threshold by both Miranda [Threshold: score(S) > 145 and free energy (ΔG) < −22 kcal/mol] and TargetScan (Threshold: context + score ≤ −0.5). Although miRNA target sites and their binding affinity may be different in different tissues, we plotted the relative precision/recall values of Miranda and Target scan results for different thresholds compared with the binding sites reported based on CLASH method (19) to see whether these thresholds would produce high quality predictions (Supplementary Figure S1a and b). Our analysis revealed that the chosen thresholds compared few other tested thresholds, are appropriate to identify relatively high precision and recall values for miRNA predictions and hence they were considered for reporting the predictions. These predictions are represented as downloadable tracks generated using UCSC genome browser in our database.

#### (I) Binding Motifs

RNA recognition capabilities of RBPs can be represented as sequence logos (20) and Position-specific Weight Matrices (PWMs) from the RNA compete experiments conducted by Ray *et al*.** (11) were obtained to generate the sequence logos for all the annotated motifs of an RBP. In addition, CLIP-seq data for 50 human RBPs was obtained from CLIPdb (21) and RBP binding peaks reported as significant by the authors were used to identify binding motifs using HOMER (22). This set was augmented by curating literature for cross-linking and SELEX data to identify additional RBPs with available motifs. All the identified binding motifs were used as PWMs to seqLogo (23) to generate sequence logos for motif representation. Each of the PWMs associated with an RBP were also scanned using Find Individual Motif Occurrences (FIMO) (24) from MEME suite (25), on all the annotated human gene sequences obtained from ENSEMBL (v73), to identify the potential binding target regions and corresponding genes. These predictions are made available as a browsable table for each motif along with the significance level of each motif instance. Only the top 4000 most significant motif occurrences from FIMO runs were included. Genes predicted to contain at least one occurrence of these significant motif instances were considered for functional enrichment analysis using g:Profiler (26). Enriched functional processes and pathways are also made available as a browsable table for each motif. For RBPs with multiple annotated motifs, each motif and its associated gene set as well as enriched processes are all accessible as a component so that users can scroll over to see the associated content for each motif.

## Results

When searched for an RBP, the following information can be identified/visualized in addition to the description, synonyms for the RBP searched (Figure 2).

Species with orthologs: This section illustrates the list of species (categorized into different taxonomic groups) in which an orthologous gene was identified to be present.

Protein Expression: This section shows the list of protein isoforms (denoted by RefSeq ids) encoded by the gene and their expression levels in 24 different tissues. Additionally, the expression levels of each isoform can be visualized as barplots.

Transcript Expression: For each protein-coding transcript encoded by the gene, we present the expression levels in 16 tissues. These expression levels measured as Reads Per Kilobase per Million mapped reads are also visualized as bar plots.

Disease Association: For each RBP, we provide the list of disease terms associated with it and the corresponding relevance score.

Protein–Protein Interactions: The physical interactions of a given RBP with other proteins is provided under this section. In addition to the gene symbol of the interactor, its corresponding synonyms, evidence code for the physical interaction which describes the experimental method used to identify the interaction and the reference to the identified interaction is also provided.

miRNA predictions: The miRNAs predicted to be targeting the 3′UTRs of the protein coding transcripts of RBPs are represented as genomic tracks. Each track image shows the transcript (ENSEMBL ID) and the binding locations of different miRNAs on its 3′UTR. MicroRNA predictions associated with each transcript of an RBP where available, are represented as downloadable tracks generated using UCSC genome browser in our database.

Binding Motifs: The sequence motif to which the RBP binds on its target genes is depicted as sequence logo and a list of target genes in the human genome which contain the top 4000 most significant occurrences of the motif are made available as a viewable table. Enriched processes and pathways corresponding to the gene list for each motif are also made available as a table for the end user.

## Future directions

We anticipate including tissue-specific protein interactions, increasing the number of RBPs with experimentally available sequence motifs as well as providing expression of RBPs in disease contexts such as in cancer samples (27) in future releases of READ DB. We believe such a unique resource on expression and disease for RBPs would not only provide a one stop portal for understanding post-transcriptional regulatory network dynamics in general but also help experimentalists working on specific RBPs to target and prioritize tissues for furthering our understanding of diverse post-transcriptional regulatory mechanisms ranging from RNA splicing to editing. We plan to update READ DB at least once per year to incorporate newly available information and to expand our repertoire of experimental conditions and tissues. As more motif data becomes available, we will build and make available more comprehensive RBP-RNA networks in a RBP-centric manner.

## Supplementary Data

Supplementary data are available at *Database* Online.

## Funding

This work was supported by School of Informatics and Computing at Indiana University Purdue University Indianapolis (IUPUI) in the form of start-up funds for SCJ. Funding for open access charge: SCJ from IUPUI.

*Conflict of interest. None declared.*

## Supplementary Material

Supplementary Data
