# Supplementary material for: Database of RNA binding protein expression and disease dynamics (READ DB)
Source: Database (Oxford). 2015 Jul 25;2015:bav072. doi: 10.1093/database/bav072 (PMC4515031; doi:10.1093/database/bav072)
Supplement: Supplementary Data [file supp_2015_bav072_index.html]

Database of RNA binding protein expression and disease dynamics (READ DB) — Supplementary Data 

# Database of RNA binding protein expression and disease dynamics (READ DB)

## Supplementary Data

files

- Supplementary Data - jpg file
