# Supplementary figures and images for: Database of RNA binding protein expression and disease dynamics (READ DB)
Source: Database (Oxford). 2015 Jul 25;2015:bav072. doi: 10.1093/database/bav072 (PMC4515031; doi:10.1093/database/bav072)

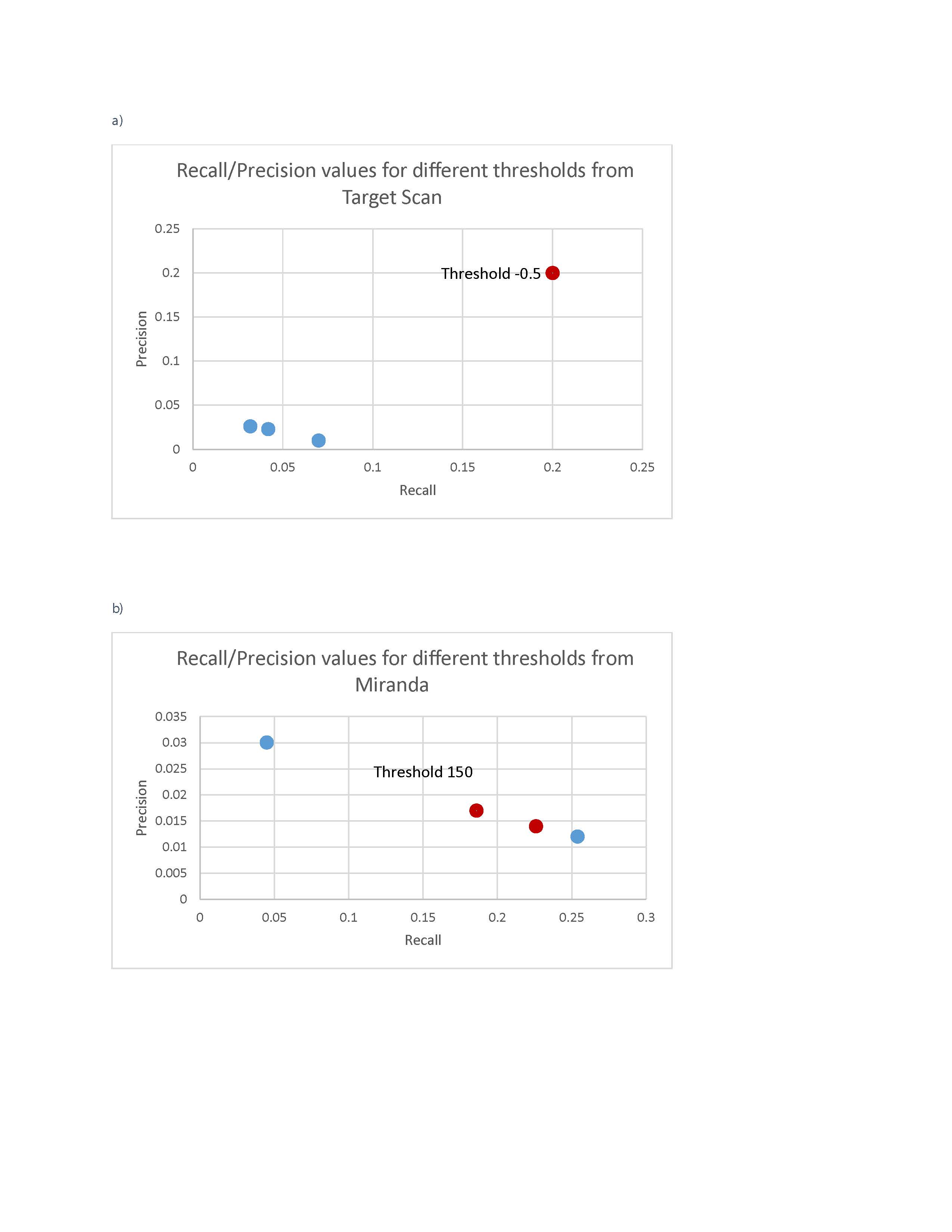

Supplement: Supplementary Data [file supp_bav072_Supplementary_Figure_1.jpg]
